# Stem cell-derived exosome treatment for acute spinal cord injury: a systematic review and meta-analysis based on preclinical evidence

**DOI:** 10.3389/fneur.2025.1447414

**Published:** 2025-01-24

**Authors:** Chunlin Mou, Ziyao Xia, Xiujuan Wang, Xunan Dai, Jiaxian Wang, Chun Zhang, Yongsheng Xu

**Affiliations:** ^1^Technology Department, Tianjin Everunion Biotechnology Co., Ltd., Beijing, China; ^2^Department of Ophthalmology, Peking University Third Hospital, Beijing, China; ^3^Beijing Tsinghua Changgung Hospital Eye Center, Beijing Tsinghua Changgung Hospital, Tsinghua Medicine, Tsinghua University, Beijing, China; ^4^Beijing Visual Science and Translational Eye Research Institute (BERI), Beijing, China; ^5^HELP Therapeutics Co., Ltd., Nanjing, China; ^6^Chongqing Institute Of Health Resources Innovation, Chongqing, China

**Keywords:** mesenchymal stem cells, exosomes, spinal cord injury, locomotor function, Basso-Beattie-Bresnahan scores, meta-analysis, spinal contusion, spinal fixation

## Abstract

**Background:**

The study aims were to systematically review and analyze preclinical research on the efficacy of exosomes derived from various mesenchymal stem cell sources (MSC-exos) for the treatment of spinal cord contusion injury (SCI) in small animal models.

**Methods:**

We conducted a systematic search of PubMed, Embase and Google Scholar databases from their inception through February 29, 2024, to identify eligible English-language studies based on predefined inclusion and exclusion criteria. Two independent investigators performed literature screening, data extraction and bias assessment.

**Results:**

A total of 235 rats were used to assess locomotor recovery at the initial assessment, and exhibited significant improvement in hind limb movement in those treated with exosomes, as indicated by a statistically significant increase in Basso-Beattie-Bresnahan (BBB) scores (MD: 1.26, 95% CI: 1.14–1.38, *p* < 0.01) compared to the controls. This trend persisted in final assessment data across 21 studies, with pooled analysis confirming similar results (MD: 1.56, 95% CI: 1.43–1.68, *p* < 0.01). Funnel plot analysis indicated asymmetry in the pooled BBB scores at both baseline and endpoint assessments, suggesting potential publication bias. Exosomes were derived from bone marrow, adipose tissue, umbilical cord or human placental MSCs. Meta-analysis results showed no statistically significant differences in therapeutic efficacy among these MSC-exos sources at various treatment time points.

**Conclusion:**

MSC-exos demonstrated considerable promise in improving motor function in SCI-affected rats, with bone marrow MSC-derived exosomes having particularly notable effectiveness.

## 1 Background

Traumatic spinal cord injury (SCI) is a severe and often life-altering condition, leading to high rates of morbidity and permanent disability worldwide (1). Each year, between 250,000 and 500,000 people experience SCI, primarily due to causes such as vehicle accidents and violence, with over 27 million individuals living with SCI-induced disabilities globally (2). Mechanistically, SCI often results from direct contusion (where external force impacts the spinal cord), hyperextension (overstretching beyond physiological limits), distraction (vertebral separation), or laceration/transection (cutting or severing of the spinal cord) (3, 4). Additionally, secondary injuries from ischemia, inflammation, and excitotoxicity exacerbate motor and sensory deficits, leading to long-term complications (5).

The standard early interventions for acute SCI primarily involve surgical approaches, such as decompression, spinal fixation and fusion, aimed at repairing initial damage and preventing spinal deformities, pain and nerve complications (6). Later-stage treatments include drugs, hyperbaric oxygen therapy and physical interventions to reduce injury progression (7). However, these treatments often fall short in delivering satisfactory outcomes for SCI patients, highlighting an urgent need for alternative therapies (8). Addressing these limitations through innovative approaches such as stem cell exosome therapy may offer improved outcomes, underscoring the necessity of advancing research in this area.

Among emerging therapies, mesenchymal stem cell (MSC)-based treatments are showing promise for SCI management (9). Numerous preclinical studies have demonstrated that stem cells have multifaceted therapeutic properties, including the ability to repair the blood-spinal cord barrier, reduce neuronal apoptosis, promote angiogenesis, regenerate axons and mitigate glial scarring and inflammation (10). Current clinical trials (phase 1 and 2) are exploring the safety and efficacy of MSCs in treating SCI, yet they also reveal adverse effects that necessitate careful consideration (11). Challenges such as low graft survival, immune rejection, genetic variability and potential tumorigenesis continue to impede the broader clinical application of stem cell therapies (12).

Recent research has highlighted the pivotal role of MSC-derived exosomes (MSC-exos) for the effectiveness of stem cell therapies (13). Exosomes – small vesicles with a phospholipid bilayer – transport specific proteins, DNA, mRNA and microRNA from stem cells to target cells, influencing cellular functions and promoting genotypic and phenotypic changes (14, 15). Functionally, exosomes enhance angiogenesis, promote axon regeneration, modulate immune responses, inhibit apoptosis and strengthen the blood-spinal cord barrier, supporting SCI repair (16). Compared to stem cells, exosomes offer advantages such as non-tumorigenicity, structural stability, low immunogenicity and improved capillary permeability, making them promising candidates for preclinical SCI research (17, 18).

Despite the growing interest in stem cell-derived exosomes for SCI therapy, the specific extent of their therapeutic benefits remains insufficiently defined in the current literature (19). The present study aims were to address this critical gap by conducting a systematic review and meta-analysis to evaluate the efficacy and feasibility of MSC-exos for SCI treatment in animal models. By synthesizing available research, this investigation provides valuable evidence to guide future studies and clinical applications, ultimately contributing to improved therapeutic strategies for individuals with SCI.

## 2 Methods

### 2.1 Search strategies and protocol

**Figure 1:**
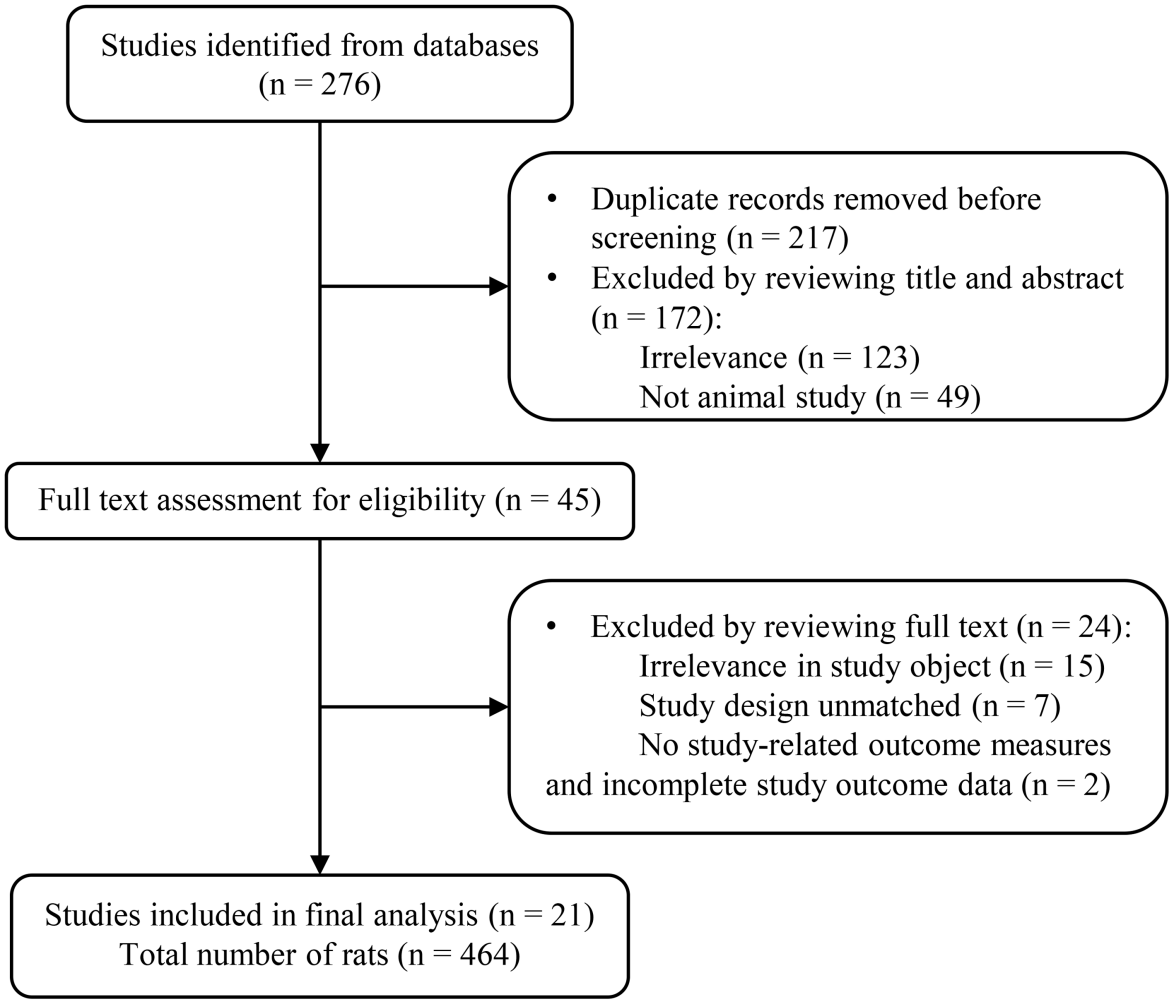
PRISMA flow chart for the article selection process.

The present meta-analysis focused exclusively on published preclinical investigations involving MSC-exos in rodent models of spinal cord contusion injury. A comprehensive search encompassed only English-language literature across prominent databases such as PubMed, Embase, Web of Science and Google scholar, spanning the period from inception to Feb 29, 2024. The search strategy was crafted to include relevant terms, such as “stem cell-derived exosomes,” “exosomes,” and “spinal cord injury” or “SCI,” within titles and abstracts. A detailed outline of the database search strategy is presented in Figure 1. Furthermore, adherence to the Preferred Reporting Items for Systematic Reviews and Meta-Analysis (PRISMA) guidelines ensured methodological rigor, with the development of a search strategy aligned with the PICOS (Population, Intervention, Comparison, Outcome, Study design) framework (20). It is worth noting that this systematic review and meta-analysis did not require ethical approval or the consent of individuals to participate.

### 2.2 Inclusion criteria

The criteria for study inclusion were: (i) only studies that utilized rat SCI models induced solely by contusion on a single vertebra were considered, aiming to mitigate heterogeneity arising from variations in animal models and SCI mechanisms, thus aligning with clinical scenarios; (ii) studies that investigated the intravenous transplantation of stem cell-derived exosomes; (iii) studies that compared the therapeutic efficacy of stem cell-derived exosomes with their respective negative controls in the context of SCI treatment being sought; and (iv) studies that provided Basso-Beattie-Bresnahan (BBB) scores for an objective assessment of motor recovery were prioritized (21). These stringent criteria ensured the selection of studies meeting high standards of methodological rigor and relevance to the research objectives.

### 2.3 Exclusion criteria

Detailed exclusion criteria were: (i) studies that involved exosomes originating from non-MSCs or those without clear exosome origin; (ii) studies that utilized animal models other than rats, such as mice, dogs, monkeys etc.; (iii) studies that employed animal models generated by spinal cord transection or ischemic-perfusion injury; (iv) studies devoid of a control group for comparison; (v) articles lacking reports on BBB scores for assessing functional recovery; (vi) studies that were systematic reviews, meta-analyses, case reports, meeting abstracts, communication letters and similar publications; and (vii) studies published in a journal with an impact factor < 1 (22). These criteria ensured the selection of studies with a high degree of relevance and methodological stringency, which enhanced the robustness of our meta-analysis.

### 2.4 Data extraction

Two independent investigators conducted a comprehensive review of the selected literature and extracted relevant data from articles meeting the predefined inclusion and exclusion criteria. The extracted data were systematically organized in an Excel spreadsheet, categorized by author, publication year, species, body weight, SCI segment, exosome origin, dosage, administration frequency, mode of administration, potential mechanism and citation details. For data presented in graphical rather than tabular form, the online platform PlotDigitizer was utilized to extract accurately relevant information.

### 2.5 Study quality assessment

Quality assessment of all included studies was conducted using SYRCLE’s tool, which provides a comprehensive evaluation of bias risk in animal studies. This tool examines ten essential domains: methodological consistency, baseline comparability, allocation concealment, housing standardization, investigator blinding, outcome randomization, assessor blinding, outcome completeness, outcome reporting consistency, and other potential biases. This thorough evaluation ensured adherence to established standards, thereby enhancing the robustness and reliability of our data.

### 2.6 Data synthesis and statistical analysis

The BBB locomotor rating scale has become a widely used tool for assessing the behavioral impact of SCI in rats, with scores ranging from 0 (no hind limb movement) to 21 (normal locomotion). Its sensitivity allows for precise differentiation of hind limb locomotor abilities across varying injury severities. Consequently, in the selected studies, improvements in locomotor function were measured using the BBB scale, focusing specifically on hind limb motor function (23). This approach ensured standardized and accurate evaluations, enabling a nuanced understanding of therapeutic effects across different SCI severities.

Data from all included studies were synthesized and analyzed using the meta-package in R software (version 4.3.1, University of Auckland, New Zealand). An initial pairwise meta-analysis was conducted to evaluate the effectiveness of MSC-exos in SCI repair, presenting all outcomes as standardized mean differences (SMDs) with 95% confidence intervals (CIs). In cases of significant heterogeneity (*p* ≤ 0.05 or *I^2^* > 50%), a random-effects model was applied; otherwise, a fixed-effects model was used. After addressing major sources of heterogeneity, the random-effects model was consistently applied for further analyses.

Using the “meta” R package, a comprehensive meta-analysis was conducted to evaluate the therapeutic efficacy of exosomes derived from stem cell sources. Fixed-effect models were applied under the assumption of a consistent true effect size across all included animal studies. Given the limited number of trials per comparison and the prevalence of single-trial evaluations, fixed-effect models were appropriate, eliminating the need to estimate between-study heterogeneity and thus enhancing the robustness of our findings.

To evaluate publication bias, funnel plots were created to visually inspect for any missing smaller studies with minimal effect sizes. Assuming no publication bias, a symmetrical distribution of studies around the pooled effect size was expected, represented by a central vertical line on the funnel plot. Asymmetry in the funnel plot indicates potential publication bias, which was quantified using Egger’s test – a statistical measure specifically designed to assess funnel plot asymmetry.

## 3 Results

### 3.1 Literature retrieval

A comprehensive keyword search across five databases yielded 276 articles. After removing duplicates, the titles and abstracts of the remaining 217 articles were reviewed, leading to the identification of 45 articles that initially appeared to meet the inclusion criteria. However, upon thorough full-text examination, the selection was refined to 21 articles that clearly met all predefined criteria. The detailed process of literature screening, with each stage of selection, is illustrated in Figure 1.

### 3.2 Baseline demographics of the included studies

The selection process yielded 21 articles, comprising a study group of 235 rats and a control group of 229 rats. The rats, aged 6 to 24 weeks, weighed between 120 and 350 g. Sample sizes within each experimental group varied from 6 to 25 rats. Across these studies, contusion was the predominant modeling method used. Exosomes derived from bone marrow, adipose tissue, umbilical cord, and human placental MSCs were used in the interventions. Bone marrow MSC-derived exosomes were featured in 12 studies, while exosomes from the other sources appeared in two studies each, except for those sourced from the umbilical cord, which were used in five studies.The therapeutic exosome dosage ranged from 100 to 400 mg, administered uniformly through the tail vein. Notably, exosome administration occurred within 12 h post-model induction. For a detailed summary of each study’s characteristics (24–44), readers are directed to view Table 1 for detailed information.

**Table 1:** Characteristics of the included studies.

#	Author	Year	Source	Administration route	Injured site	Modeling method	Possible mechanism	Reference
1	Afsartala et al.	2023	Adipose tissue mesenchymal stem cell	Tail vein injection	T9–10	Contusion	N/A	(24)
2	Chang et al.	2021	Bone marrow mesenchymal stem cell	Tail vein injection	T10	Contusion	M2 macrophage polarization in spinal cord injury by downregulating IRF5	(25)
3	Chen et al.	2021	Bone marrow mesenchymal stem cell	Tail vein injection	T10	Contusion	Promoting axonal regeneration via the PTEN/AKT/mTOR pathway	(26)
4	Cheshmi et al.	2023	Human placental mesenchymal stem cell	Tail vein injection	T9	Contusion	N/A	(27)
5	Huang et al.	2019	Bone marrow mesenchymal stem cell	Tail vein injection	T10	Contusion	Silencing CTGF gene	(28)
6	Huang et al.	2021	Bone marrow mesenchymal stem cell	Tail vein injection	T10	Contusion	Promoting neurofilament regeneration	(29)
7	Jiang et al.	2021	Bone marrow mesenchymal stem cell	Tail vein injection	T10	Contusion	Reducing inflammation in spinal cord injury by regulating the TLR4/NK-κB signaling pathway	(30)
8	Kang et al.	2022	Human umbilical cord mesenchymal stem cell	Tail vein injection	T10	Contusion	N/A	(31)
9	Liu et al.	2022	Bone marrow mesenchymal stem cell	Tail vein injection	T9–10	Contusion	N/A	(32)
10	Liu et al.	2020	Bone marrow mesenchymal stem cell	Tail vein injection	T10	Contusion	Shifting microglial M1/M2 polarization	(33)
11	Mu et al.	2021	Human umbilical cord mesenchymal stem cell	Tail vein injection	T9–10	Contusion	N/A	(34)
12	Nakazaki et al.	2021	Bone marrow mesenchymal stem cell	Tail vein injection	T9	Contusion	Promoting TGF-*β* upregulation, microvascular stabilization	(35)
13	Romanelli et al.	2019	Human umbilical cord mesenchymal stem cell	Tail vein injection	T8	Contusion	N/A	(36)
14	Shao et al.	2023	Bone marrow mesenchymal stem cell	Tail vein injection	T10	Contusion	N/A	(37)
15	Sung et al.	2022	Human adipose tissue mesenchymal stem cell	Tail vein injection	T9	Contusion	N/A	(38)
16	Wang et al.	2021	Human umbilical mesenchymal stem cell	Tail vein injection	T9	Contusion	Upregulation of NGF/TrkA signaling pathway	(39)
17	Wang et al.	2018	Bone marrow mesenchymal stem cell	Tail vein injection	T10	Contusion	Downregulation of phosphorylated NFκB P65 subunit	(40)
18	Xue et al.	2023	Human umbilical cord mesenchymal stem cell	Tail vein injection	T10	Contusion	Down-regulation of Endothelin-1	(41)
19	Zhang et al.	2021	Bone marrow mesenchymal stem cell	Tail vein injection	T10	Contusion	N/A	(42)
20	Zhou et al.	2022	Bone marrow mesenchymal stem cell	Tail vein injection	T10	Contusion	Inhibiting pericyte pyroptosis	(43)
21	Zhou et al.	2021	Human placental mesenchymal stem cell	Tail vein injection	T11	Contusion	Activating endogenous neurogenesis	(44)

The 21 studies on MSC-exos treatment in acute spinal injury revealed a range of intricate and diverse therapeutic mechanisms. These included modulating M2 macrophage polarization within the SCI environment through IRF5 downregulation and promoting axonal regeneration via the PTEN/AKT/mTOR pathway, with further therapeutic benefits from CTGF gene silencing. Notable actions also encompassed neurofilament regeneration, reducing inflammation by modulating the TLR4/NF-κB signaling pathway, and balancing microglial M1/M2 polarization. Additionally, exosomes enhanced TGF-*β* upregulation, supporting microvascular stability and activated the NGF/TrkA signaling pathway to promote neurotrophic support. Other critical mechanisms included downregulation of phosphorylated NFκB P65 and endothelin-1, which inhibited pericyte pyroptosis, and stimulation of endogenous neurogenesis. Collectively, these findings underscored the robust therapeutic potential and multifaceted modulatory effects of MSC-exos in SCI treatment.

### 3.3 Comparison of BBB scores between exosome-treated and control rats

**Figure 2:**
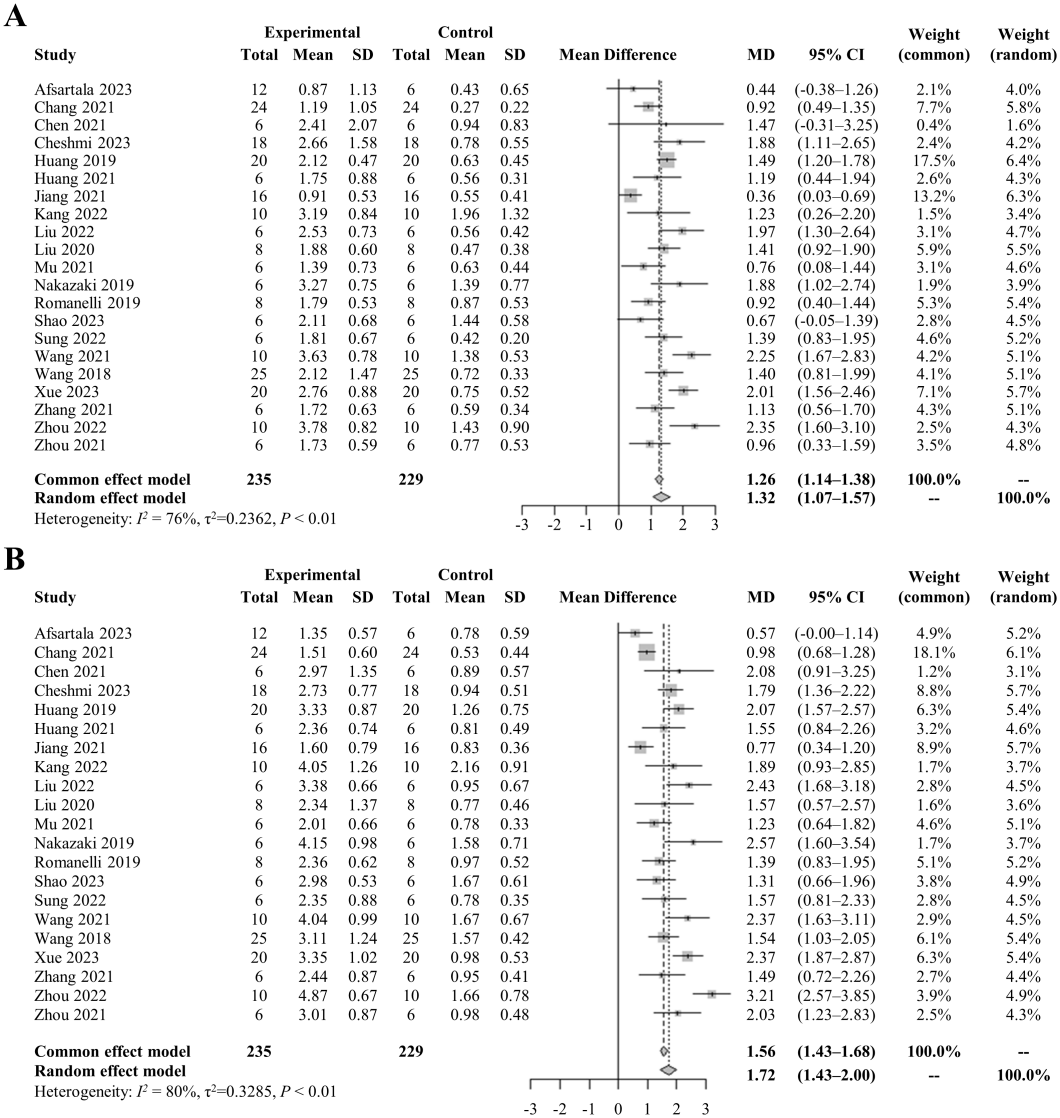
**(A)** Pooled-analysis of Basso, Beattie, and Bresnahan scale at the first measurement after SCI. **(B)** Pooled-analysis of Basso, Beattie, and Bresnahan scale at the last measurement after SCI. CI, confidence interval; MD, mean difference; SCI, spinal cord injury; SD, standard difference.

**Figure 3:**
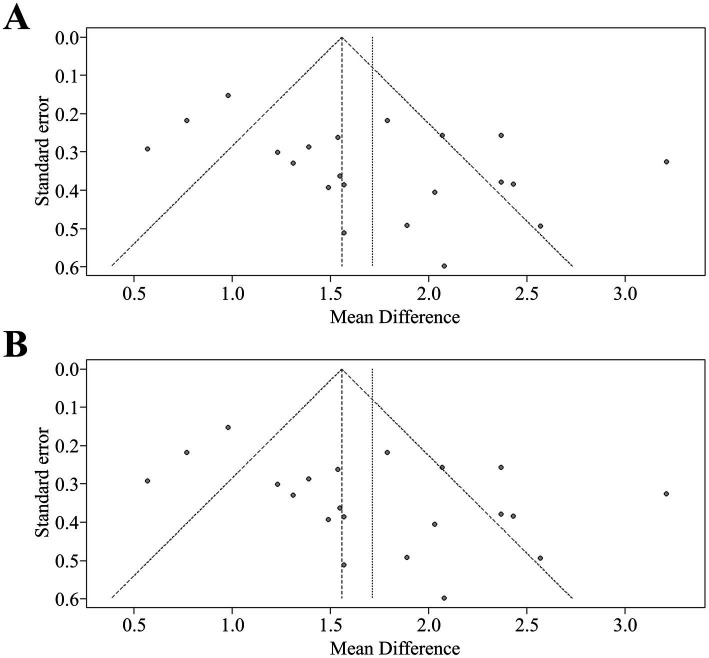
**(A)** Funnel plot for asymmetry of pooled analysis of BBB at the first measurement. **(B)** Funnel plot for asymmetry of pooled analysis of BBB at the last measurement. BBB, Basso-Beattie-Bresnahan scale.

A comprehensive analysis of all studies was conducted, involving a total of 235 rats, that reported locomotor recovery outcomes at initial assessment. The findings showed a significant improvement in hind limb movement in exosome-treated rats, as indicated by a statistically significant increase in BBB scores (mean difference: 1.26, 95% CI: 1.14–1.38, *p* < 0.01) compared to control rats at the first measurement (Figure 2). This trend continued in the final assessments, reported in 21 studies, with pooled analysis confirming a similar result (mean difference: 1.56, 95% CI: 1.43–1.68, *p* < 0.01) (Figures 2A,B). To visualize data distribution and assess potential publication bias, funnel plots were created for the pooled BBB scores at both initial and final measurements (Figures 3A,B). These analyses reinforced the robustness of the findings while underscoring the importance of considering potential biases in interpreting the results.

### 3.4 Bias of included studies

**Figure 4:**
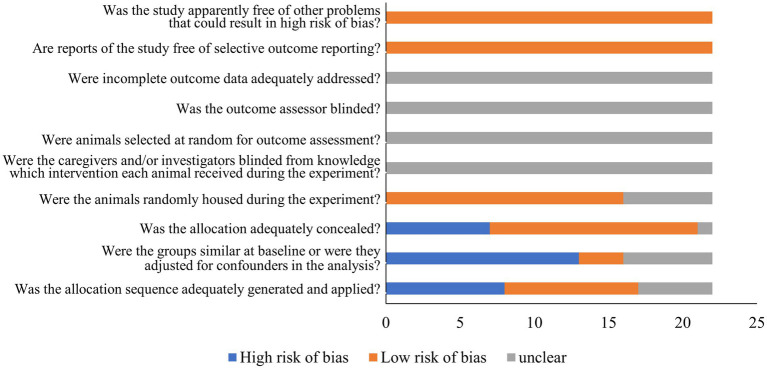
Results from the SYRCLE risk of bias tool.

Using SYRCLE’s comprehensive evaluation tool, the quality of the included articles. Our analysis was assessed and revealed that most studies adhered strongly to standards of randomization and blinding protocols, with only a few reporting issues in either randomization or blinding. Notably, other potential biases were minimal across the reviewed literature. To provide a well-rounded view of potential publication biases, the findings are visually presented in Figure 4. This evaluation demonstrates the methodological rigor of the included studies while also identifying biases that should be considered when interpreting the present results.

## 4 Discussion

Exosomes derived from various stem cell sources have gained prominence in experimental SCI treatment due to their ability to overcome limitations inherent in traditional stem cell therapies (45). In this comprehensive analysis of 21 selected studies, the efficacy of exosomes compared to placebo was rigorously evaluated. The results revealed a significant improvement in motor function in SCI-afflicted rats treated with MSC-exos across multiple time points. Notably, the therapeutic effects of MSC-exos were most pronounced within the first four weeks post-administration, indicating sustained and robust efficacy. This result suggested a potential mechanism for their prolonged therapeutic impact – possibly due to their extended presence at the SCI site. These findings also indicate the promise of exosome-based therapies for SCI management and provide valuable insights into the temporal dynamics of their effects, guiding future research and clinical translation efforts.

Recent findings align with two prior meta-analyses that explored the therapeutic potential of stem cell-derived exosomes in rodent models of SCI. Yi and colleagues conducted a systematic review of studies up to January 2021, covering 35 articles with 699 rodents. Their analysis showed significant motor function improvement in SCI rodents treated with stem cell-derived exosomes, with slower recovery observed in compression injuries compared to contusion and transection injuries post-exosome therapy (46). Similarly, Shang et al. conducted a network meta-analysis of pooled data from 40 preclinical studies through March 2023, focusing on the efficacy of stem cell-derived exosomes in SCI rat models; however, they found no significant differences in therapeutic outcomes across various MSC-exos at different treatment time points (47). Unlike these studies, the present meta-analysis concentrated on rat models of SCI induced specifically by contusion, reflecting the most common clinical scenario characterized by maximal irreversible neurological deficit. To strengthen the findings, included only studies using a single-vertebral injury model were included, thereby reducing potential heterogeneity and enhancing the relevance of the results.

Numerous preclinical studies have highlighted the promising therapeutic potential of stem cell-derived exosomes for treating SCI (48). However, before advancing this cell-free, exosome-based therapy to clinical trials, critical questions regarding therapeutic efficacy and safety must be thoroughly addressed. Chief among these concerns is identifying the optimal exosome source, as exosomes from different stem cell origins exhibit varying therapeutic potentials. To date, experimental SCI treatments have primarily used exosomes derived from various MSC sources, including bone marrow, umbilical cord, and adipose tissue (49). Notably, no studies have directly compared the therapeutic efficacy of exosomes from these different stem cell sources. To address this gap, a network meta-analysis was employed to assess comprehensively the therapeutic potential of exosomes from diverse origins. Our analysis found no statistically significant differences in SCI improvement efficacy among exosomes from the four distinct sources. While these findings align with the meta-analysis by Shang et al., they contrast with Liu et al., who suggested that umbilical cord-derived MSCs might be an optimal source, citing their availability and ethical advantages. Importantly, indirect comparisons across separate studies cannot serve as definitive evidence for the differential efficacy of exosomes from various sources in SCI treatment (50). Further rigorous research, including direct comparative studies, is essential to gain conclusive insights into this critical aspect of exosome-based therapy.

While existing evidence highlights the therapeutic potential of stem cell-derived exosomes in SCI repair, significant challenges remain before clinical application is feasible. Foremost, the complex molecular mechanisms underlying exosome synthesis, secretion and cellular uptake are not yet fully understood, warranting further research (51). Additionally, the methods for isolating, purifying, identifying and scaling exosome production need refinement, as recent studies have suggested (52). Precise quantification of MSC-exos and a deeper understanding of biological modifications to enhance their therapeutic efficacy are essential areas for ongoing investigation (53). Selecting optimal administration methods to improve both the efficacy and safety of MSC-exos is another critical area for innovation (54). Preclinical studies remain indispensable for evaluating MSC-exos’ effectiveness in SCI repair, identifying key therapeutic targets and pathways, and optimizing exosome sources (55).

Addressing the limitations inherent in the present study is essential. The predominance of positive results across studies raises concerns about potential publication bias, as negative findings may be concealed or unpublished, leading to potentially misleading conclusions. Additionally, the fundamental differences between animal studies and randomized clinical trials pose challenges in collecting detailed data for each experimental group; key details such as SCI severity, model dosage and administration methods were frequently missing, complicating data synthesis and interpretation. Variations in MSC-exos dosages and administration frequencies across studies introduce further bias, as these factors significantly influence therapeutic outcomes but are inconsistently standardized or reported. Differences in exosome extraction methods also affect exosome composition and efficacy, adding another layer of variability that hampers result comparability. Ambiguities and incomplete data were common; some studies reported only exosome volume or concentration, while four did not specify the injured spinal cord segment. Small sample sizes, particularly in studies involving mice, necessitate caution in interpreting locomotor recovery outcomes. Additionally, subjective interpretation introduces variability, as outcomes may be affected by observer bias and blinding status. Consequently, while the present findings provide valuable insights, translating them into actionable conclusions must be approached with due caution and consideration of these limitations.

In conclusion, MSC-exos show substantial therapeutic efficacy in improving motor function in rats with SCI, with bone marrow MSC-exos exhibiting particularly notable potential. However, reliance on animal studies, which are generally lower in evidence quality, introduces some uncertainty regarding the robustness and reliability of these findings. This highlights the need for high-quality, direct comparative studies to clarify the nuances of exosome-based therapy, identify the optimal stem cell sources, and strengthen confidence in its translational potential for SCI management.

## Data Availability

The original contributions presented in the study are included in the article/supplementary material, further inquiries can be directed to the corresponding authors.
